# Toward neuroprosthetic real-time communication from *in silico* to biological neuronal network via patterned optogenetic stimulation

**DOI:** 10.1038/s41598-020-63934-4

**Published:** 2020-05-05

**Authors:** Yossi Mosbacher, Farad Khoyratee, Miri Goldin, Sivan Kanner, Yenehaetra Malakai, Moises Silva, Filippo Grassia, Yoav Ben Simon, Jesus Cortes, Ari Barzilai, Timothée Levi, Paolo Bonifazi

**Affiliations:** 1 Department of Particle Physics and Astrophysics, Weizmann Institute of Science, Rehovot, Israel; 2 IMS laboratory, CNRS UMR 5218, University of Bordeaux, Talence, 33405 France; 3 Department of Neurobiology, George S. Wise, Faculty of Life Sciences, Tel Aviv University, Tel Aviv, 69978 Israel; 4 GEII department, IUT Bordeaux, University of Bordeaux, Gradignan, 33175 France; 5 Faculty of Informatics, University of the Basque Country, San Sebastian, 20018 Spain; 6 Laboratory of Innovative Technologies, University of Picardie Jules Verne, Amiens, 80025 France; 7 Department of Cell Biology and Histology, University of the Basque Country, Leioa, 48940 Spain; 8 Sagol School of Neuroscience, Tel Aviv University, Tel Aviv, 69978 Israel; 9 IKERBASQUE, The Basque Fundation, Bilbao, 48013 Spain; 10 Biocruces Health Research Institute, Barakaldo, 48903 Spain; 11 LIMMS/CNRS-IIS, The University of Tokyo, Tokyo, 153 8505 Japan; 12 IIS, The University of Tokyo, Tokyo, 153-8505 Japan; 13 School of Physics and Astronomy, Tel Aviv University, Tel Aviv, 69978 Israel; 14 Friedrich Miescher Institute for Biomedical Research, University of Basel, Maulbeerstrasse 66, Basel, 4058 Switzerland

**Keywords:** Neurology, Electrical and electronic engineering, Optical techniques

## Abstract

Restoration of the communication between brain circuitry is a crucial step in the recovery of brain damage induced by traumatic injuries or neurological insults. In this work we present a study of real-time unidirectional communication between a spiking neuronal network (SNN) implemented on digital platform and an *in-vitro* biological neuronal network (BNN), generating similar spontaneous patterns of activity both spatial and temporal. The communication between the networks was established using patterned optogenetic stimulation via a modified digital light projector (DLP) receiving real-time input dictated by the spiking neurons’ state. Each stimulation consisted of a binary image composed of 8 × 8 squares, representing the state of 64 excitatory neurons. The spontaneous and evoked activity of the biological neuronal network was recorded using a multi-electrode array in conjunction with calcium imaging. The image was projected in a sub-portion of the cultured network covered by a subset of the all electrodes. The unidirectional information transmission (SNN to BNN) is estimated using the similarity matrix of the input stimuli and output firing. Information transmission was studied in relation to the distribution of stimulus frequency and stimulus intensity, both regulated by the spontaneous dynamics of the SNN, and to the entrainment of the biological networks. We demonstrate that high information transfer from SNN to BNN is possible and identify a set of conditions under which such transfer can occur, namely when the spiking network synchronizations drive the biological synchronizations (entrainment) and in a linear regime response to the stimuli. This research provides further evidence of possible application of miniaturized SNN in future neuro-prosthetic devices for local replacement of injured micro-circuitries capable to communicate within larger brain networks.

## Introduction

Neurological disorders and traumas impact the structural and functional properties of brain networks and circuits, causing cell death, loss of synapses, and loss of axons projecting locally within microcircuits or distantly to other brain regions. These effects impair local information processing capabilities and information exchange between distant circuitry, disrupting the process of segregation and integration of information in the brain^1^.

In this context, new therapeutic approaches and technologies are needed both to promote cell survival and regeneration of local circuits with the integration of new neuro-glial cells^2,3^, as well as to restore long distance communication between disconnected brain regions and circuits^4,5^.

While cellular therapies have been shown promise in engrafting and refilling circuitries^2,3^, regenerating lost long-distance connections seem to be more challenging, since these are early developed circuitry architectures which are difficult to reprogram and recreate^6,7^.

Contrastly, major progress has been made in the field of neuro-prosthesis^6,8^ over the past decade, where artificial spiking neural circuits are locally capable of receiving and processing input in real time while their output can be delivered locally or remotely, either through electrical or optogenetic stimulation^9,10^. This enables fast, bi-directional control of multiple cell types^11^.

Various approaches exist in the field of neuromorphic engineering to the design of Spiking Neural Network (SNN) and artificial synapses. In the case of neuro-inspired axis, the SNN is quite distinct from biological activity and was designed mostly for applications such as computation and artificial intelligence. The neuromimetic axis on the other hand, imitates more precisely the activity of neuronal cells and operate at accelerated or biological time scales. This SNN can be simulated by software^12–14^ but real-time simulation is difficult to achieve, making it unsuitable for bio-hybrid experiments. Hardware SNNs work in real-time, are low power consumption and embedded, a promising choice for hybrid experiments and new generation neuroprosthesis. Hardware SNN^15–21^ can be classified in two groups: analog implementation and digital implementation. The digital implementation has the advantage to being tunable and easier to process, despite its higher power consumption. Artificial synapses for spike processing and for bio-physics interface provide biomimetic solutions. Memristive devices can process neural spikes and emulate synapses^22,23^. Efficient real-time data compression with low energy is possible thanks to these memristor-based systems.

To create bioelectrical therapeutic solutions for health care, a real-time bio-physics interface is crucial^24^. Few experiments have succeeded in building a real-time system which can provide adaptive stimulation using SNN^4,25,26^.

In this paper, we present new evidence of real time communication and information transfer from a hardware SNN implemented on an FPGA board and an *in-vitro* biological neuronal network (BNN), by real-time encoding dynamics of a SNN in patterns used for optogenetic stimulation of the BNN.

Biologically-inspired patterns of activity were generated using the SNN and then encoded in real-time into unique patterns of blue light (binary images of 8 × 8 large pixels generated via digital light processing (DLP) using a modified video projector micro-projected on to a 2D neuronal network (culture) grown on a multi-electrode array (MEA). Neurons, which were transduced using an Adeno-associated virus (AAV) to express the fast Channelrhodopsin2 (ChR2) variant ChIEF^27^. The neurons were therefore excited by the blue light stimulation, and their activity was recorded both by the MEA device and through calcium imaging using a fast electron multiplying charge coupled device (EMCCD) camera mounted on the microscope. The area which was optically stimulated covered a limited portion of the cultured network of about 0.8 × 0.8 mm, out of a global network area of several squared millimetres. Therefore, communication and information transfer were tested on a subnetwork of the BNN which was also monitored by about one fourth (4 × 4) of the total number of monitoring electrodes (8 × 8). Indeed, the interaction between global BNN dynamics (such as spontaneous synchronizations) and local information transfer (occurring in the stimulated subnetworks) is a key point of the results presented in this work.

The experimental data was analysed in relation to the different parameters which were varied in the SNN (concerning spontaneous dynamics and output conversion) and the intrinsic dynamics of the biological neuronal network (BNN), driven internally by spontaneous network burst^28^. Results show that in some optimal conditions, where the SNN activity is capable to entrain the BNN activity and suppress spontaneous synchronizations, and in a linear regime of BNN response, information transmission, measured as similarity between INPUT and OUTPUT patterns, can occur.

These results provide further evidence of possible application of miniaturized SNN in future neuro-prosthetic devices for local replacement of injured micro-circuitries (restoring local information processing or segregation) capable to communicate within larger brain networks.

## Results

### The set-up

**Figure 1:**
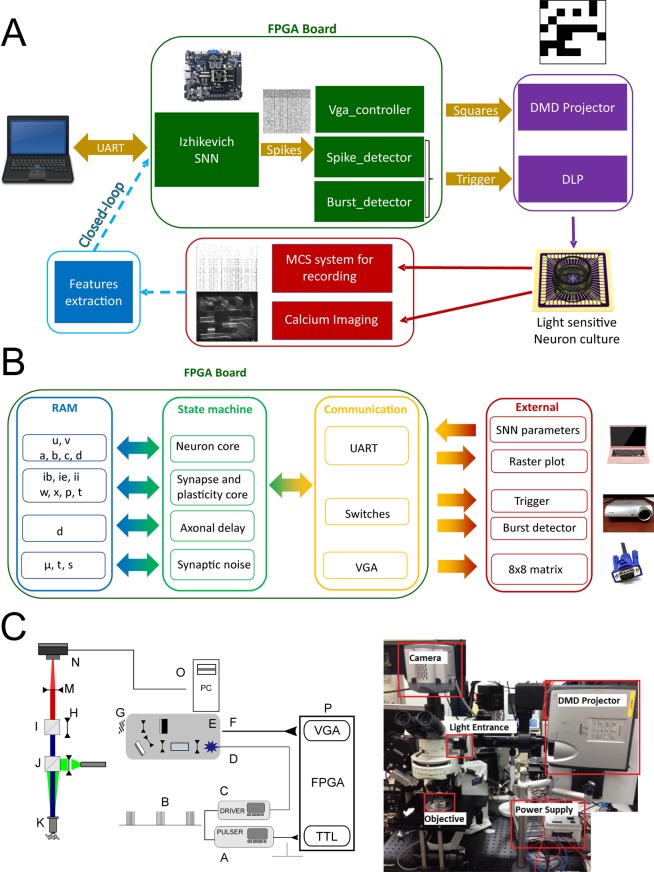
Scheme of the experimental set-up. (**A**) The experimental set-up is composed by the FPGA board on which the spiking neural network is running, the video-projecting system (a Digital Micromirror Device-projector (DMD)) organized around an upright microscope (right image) and the biological neuronal network (culture) grown on a multi-electrode array. Neural activity is monitored by electrical recordings and calcium imaging. The perspectives of this set-up is to add a real-time features extraction from the biological neuronal network to stimulate the spiking neural network, in order to achieve a real-time bi-directional communication between the two networks. (**B**) Neuromorphic board schematic block. FPGA board includes different VHDL modules: SNN core (neuron, synapse, plasticity, axonal delay and synaptic noise), UART, VGA and switch communication, burst detector module. All parameters are stored in RAM and are updated every 1 ms. The burst detector module sends triggering signal to the STG stimulator and enable signal for 8 × 8 matrix image. The SNN core module sends raster plots updated at a defined interval via UART and neuron activity for VGA image module. (**C**) Left Scheme of the optical path and TTL-control of the video-projection. The FPGA board (P) sends two simultaneous output: the VGA video signal (F) to the video-projector (E, Modified Sharp Notevision XR-10X DMD) coding for the image representing the 8 × 8 binary matrix shaped by the SNN activity, and the TTL signal triggering the stimulator (A). The stimulator generate the signal (B) with 5 pulses of 5 V lasting 30 ms with an interval of 40 ms which control the custom-made power supply of the Luminus PT-120-B High Power blue LED (D). The image generated by the video-projector (G) is at the focal length (250 mm) of the coupling lens (H) located in front of the cube (I) with the long-pass dichroic mirror and the emission filter (located above the cube). The other cube (J) contains the dichroic mirror and excitation notch filter (dsRed) for red calcium imaging. The image (G) is focused at the adjustable stage (L) of the microscope (L) through a 10x objective (K). The sample image located at the stage (L) is recorded by the camera mounted on the microscope (N) after being focused by the tube lens M. A PC (O) recorded the camera images. (C) Right Picture of the optical set-up including DMD projector which projects into an up-right epifluorescence microscope through an additional optical pathway obtained in between the camera and the excitation/dichroic cube placed above the neuron culture, by splitting orthogonally the camera pathway with a dichroic mirror.

The experimental set up (Fig. 1A) is built out of three main components arranged around an up-right epifluorescent microscope adapted to perform the experiments described in this paper. Specifically: (1) the spiking neural network (SNN) (Fig. 1B) running on a FPGA board (i.e. a real-time digital electronic board which is embedded and low-power), (2) the system for video-projecting the SNN-FPGA image-output (delivered to digital light projector through a video port (Fig. 1C)) and (3) the biological neuronal network expressing ChIEF-mCitrine (under control of the human synapsin promoter, hSyn) which enables optogenetic blue-light activation of the neurons, grown on a multi-electrode array placed at the focal plane of the microscope (where stimuli images are video-projected). The three components and their functional organization in relation to the set-up and the global objective of this work, are separately described below.

### SNN: spontaneous dynamics and output image generation

In order to simulate the activity of a real BNN, the SNN used in this study^4,29^ generated spontaneous activity characterized by neuronal synchronizations with similar features (in terms of duration, frequency and number of recruited neurons) to those generated by the cortical BNNs used in this work, which is typically in biology between 0.1 to 1 Hz^28^.

The four different SNNs used in the 12 experiments presented in this research (see Table 1) were composed by 100 Izhikevich neurons^30^ (80 excitatory and 20 inhibitory) implemented in a FPGA (see Table 2) and allowed a range of dynamics with network synchronizations (NSs) which spanned from 0.25 to 1 Hz^4^ (see Table 3).

**Table 1:** Parameters of SNN, neuron parameter includes a,b,c,d Izhikevich neuron parameters and x, P, τ synapses parameters, defined in Eqs. (1) to (4) in method section.

	SNN1	SNN2	SNN3	SNN4
Neurons	100	100	100	100
Connectivity (%)	25	25	25	27
Neuron parameter	Set A	Set B	Set B	Set B
Mean w_exc_	1	1	1	1
Mean w_inh_	−2	−2	−1	−1

**Table 2:** FPGA resources. LUT and FF are basic components of logic blocks into the FPGA.

Resource	Utilization	Percentage
LUT	17967	8.82
LUTRAM	660	1.03
FF	9355	2.30
BRAM	51	11.46
DSP	40	4.76

LUTRAM and BRAM are memory technologies. DSP (Digital Signal Processing) are circuits used for complex digital computation like multiplication.

**Table 3:** Experiment parameters.

Experiments	SNN	Threshold value N	Duration of NS computation (ms) T	NS by minute
1	SNN2	10	200	61
2	SNN2	10	200	55
3	SNN1	10	200	15
4	SNN1	10	200	15
5	SNN4	10	100	34
6	SNN3	10	100	21
7	SNN1	10	200	16
8	SNN3	4	100	41
9	SNN3	4	100	41
10	SNN3	4	100	41
11	SNN3	4	100	38
12	SNN3	7	100	31

For each experiment, one SNN between the four, the threshold value T of spiking neurons and time windows duration T for network synchronization computation are chosen. The number of network synchronization (NS) is given as in indicator. NS by minute is an average value of NS for the whole experiment (around 5–6 min by experiment).

SNN generated activity with a temporal resolution of 1 ms (see the raster plot of the SNN2 in sup. Fig. 1) and NSs were defined when at least N neurons of the 64 neurons spiked in a time bin B (see Table 3). The four SNNs present different activities as their neurons, synapses and connectivity parameters changed across experiments (see Methods for definition of the parameters and Table 1 for used values).

As described in suppl. Fig. 2, the spontaneous activity of the SNN was converted in real-time into binary matrices of 8 × 8 dimension, where each element of the matrix was zero (no light) if its correspondent assigned spiking neurons were not firing, or one (light emission) if the neurons were firing. Once a NS was identified, the correspondent converted image was shined. Based on the SNN activity, a network synchronization detector module created an enable Transistor-Transistor Logic (TTL) signal to the stimulator device and generate the shine of the VGA image corresponding to the 8 × 8 matrix (see Methods, Fig. 1B and Suppl. Fig. 2).

We implement the whole system with SNN, Burst detector, neuron activity trigger, data transmission through UART and video-projector handle using VGA functions in an FPGA development board (Genesys 2 with a Kintex-7 FPGA). The whole resource is just 10% of the FPGA (Table 2) and then allow in the future to integrate the feature extraction module for real-time closed-loop (Fig. 1A).

#### Video-projection system

The 8 × 8 binary matrix image generated as an output by the SNN activity, was converted into an 800 × 600 pixel image through the VGA input port of a video-projector where a high-power blue LED replaced the original light bulb. The binary 8 × 8 matrix (zero = black, one=blue) was displayed in central 600 × 600 pixels, other pixels were set to zero (black).

The image generated by the Digital Micromirror Device (DMD) of the video projector was projected into an up-right epifluorescence microscope through an additional optical pathway obtained in between the camera and the excitation/dichroic cube placed above the sample, by splitting orthogonally the camera pathway with a dichroic mirror (see Fig. 1C). The focusing of the DMD image on the microscope port was optimized to be able to project the all generated images within the field of the view of the microscope through a 10x magnification objective, with a sufficient power to evoke action potentials in neurons expressing ChIEF (see Methods; neurons were transfected at least two weeks before the experiment). The projected DMD image was located at the focal plane of a lens with a focal distance of 250 mm, de-magnifying the image by a factor of roughly fourteen times.

#### BNN: stimulation, recording and inter-device synchronization

**Figure 2:**
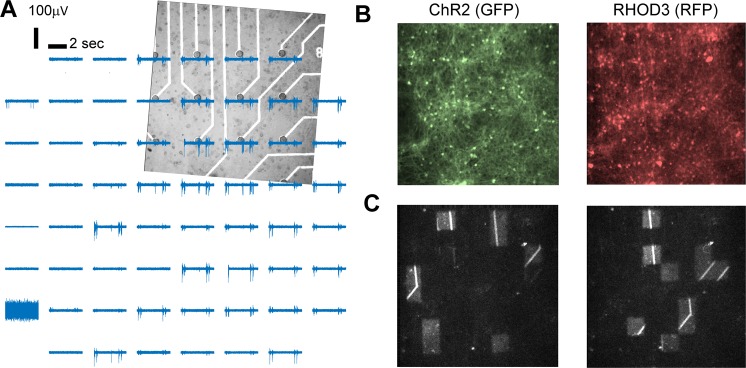
MEA recordings, calcium imaging and video-projection of images. (**A**) Bright field image of the neuronal culture grown on the MEA corresponding to the area where images (stimuli) from the SNN were projected. Binary images (blue and black) were projected. The blue light (460 nm) was triggering the response of light sensitive neurons, expressing the ChR2 variant CHiEF. (**B**) Expression of ChR2 (left) in the same area shown in (**A**) The activity of the neurons was also recorded with the red calcium sensor RHOD-3 (right). (**C**) Two representative blue light patterns of stimulation (black and white image).

Neuronal cultures between 21 DIV to 28 DIV were used (see Methods). The activity of the neurons was recorded using a standard 8 × 8 MEA dish with an inter-electrode distance of 200 micrometers (Fig. 2A). Excitatory and inhibitory neurons were transduced (under the hSyn promoter, see Methods) at 7 DIV in order to express ChIEF-mCitrine (Fig. 2B). The rate of expression in the whole neuronal population at 21 DIV was 70 +/− 13% (GFP-positive versus NeuN-positive cells on N = 6 cortical cultures; see Methods). Calcium imaging movies (with the RFP sensor RHOD-3, see Methods, Fig. 2B) were captured using a 10x objective magnification by a fast EMCCD camera mounted on the microscope, with a field of view of 800 × 800 micrometers, i.e. about the space in between 4 × 4 electrodes of the MEA (Fig. 2A). The projected image (i.e. stimulus) was applied in the same field of view but on a slightly smaller area (Fig. 2C). The activity of the neurons was also recorded through calcium imaging (using the RFP indicator, RHOD-3) using an ECCD camera (Sup. Fig. 3). Filters and dichroic mirrors of the original RFP cube have been adapted to be able to perform simultaneously ChIEF stimulation with blue light and RFP calcium imaging as described in the methods. The video-projected images of stimulations were also captured by the camera, as residual artefact blue light capable to pass through dichroic and emission filters (Fig. 2C and Sup. Fig. 3).

The synchronization between the time of the different devices (Sup. Fig. 4A), has been made through the MEA acquisition system where the signal from each of the 60 electrodes has been recorded simultaneously to the TTL signal activating the stimulation protocol controlling the LED driver (switching ON and OFF the blue light) and the to the single frame signal acquired by the camera.

Note that the MEA system was acquiring the activity of the BNN before, during and after the video-projection stimulation was switched ON (i.e. when the potential communication between the SNN and the BNN was activated, Sup. Fig. 4B). Also, the camera was independently acquiring images for calcium imaging (at 57 Hz) when the video-projection stimulation was running (Sup. Fig. 4B), so light stimulation patterns were captured as artefacts in the recorded calcium signals (Suppl. Fig. 3 and Fig. 2C).

### Spiking neuronal network

In this work we present the results from 12 different sessions of communication between the SNNs and the BNNs (Sup. Fig. 5). In each of the sessions, different SNN and thresholds for Network Synchronization (NS) detection were used to enlarge the range of stimuli by minute. This is summarized in Table 3.

**Figure 3:**
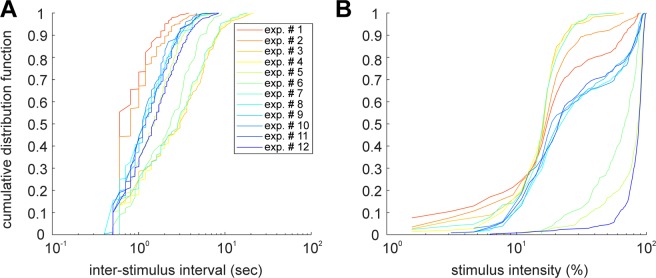
Inter-stimulation interval and intensity distribution of the stimuli generated by the SNN. Across the experiments the parameters regulating the spontaneous SNN dynamics were slightly changed in order to observe their impact on the coupling BNN and the SNN.

Varying the parameters, the SNN was generating different OUTPUTs with different ranges of frequency (measured as inter-stimulus interval, Fig. 3A) and intensity (100% stimulus intensity corresponded to all the 64 squares of the 8 × 8 matrix turned ON, Fig. 3B).

From our library of SNNs^4^ which generate spontaneous activity characterized by neuronal synchronizations with similar features to those generated by the cortical BNNs, we chose four SNNs with different characteristics, especially in network synchronization (NS) frequency. Here, the SNN NS frequency interval is [0.25; 1] Hz. This choice is due to avoid overlap stimulations as the stimulation protocol lasts 310 ms and as our cortical BNNs generate an average neuronal synchronization between 0.1 to 1 Hz^25^.

### INPUT-OUTPUT similarity and information transmission

**Figure 4:**
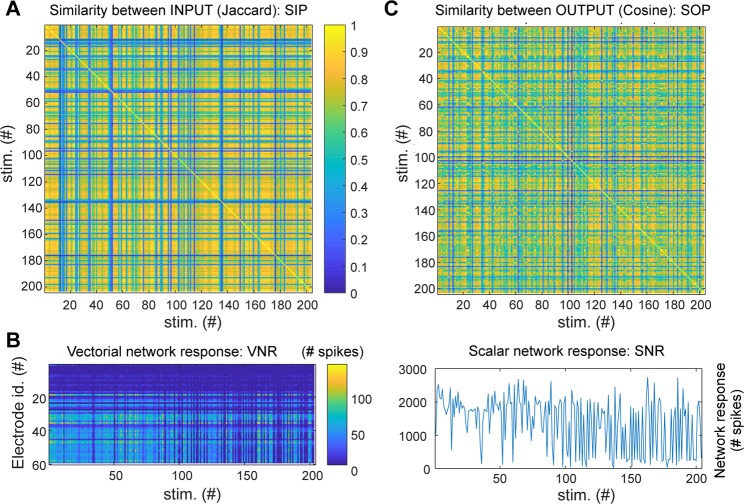
Definition of INPUT and OUPUT: computing vectorial, scalar network responses and similarity matrices for INPUT and OUTPUT. (**A**) Similarity between 8 × 8 INPUT images for a representative experiment where about 200 stimuli were projected. The Jaccard coefficient (which quantifies the intersection over the union of the ensembles of illuminated pixels of the 8 × 8 images) was used as a measure of similarity between INPUT pairs. Note that blue strips in the matrix correspond to stimuli composed by very few bright squares out of the 8 × 8 (typically less than ten) so their probability to be reproduced across time was very low (poor repetition) and their similarity to stimuli with many squares was also low (cardinality of intersection ensemble is small compared to cardinality of union ensemble, see Methods). (**B**) The VNR (left) of the BNN was computed counting for each electrode the number of spikes in the time window T (varying between 10 and 500 ms) following the stimulus. The SNR (right) is the sum of the VNR over all electrodes. Note that varying the time window T, where the network response is calculated, affects the VNR, SNR and SOP. The rationale and the impact of the choice of T is provided in relation to the description of Fig. 5C. (**C**) Similarity between VNR calculated with the cosine distance.

**Figure 5:**
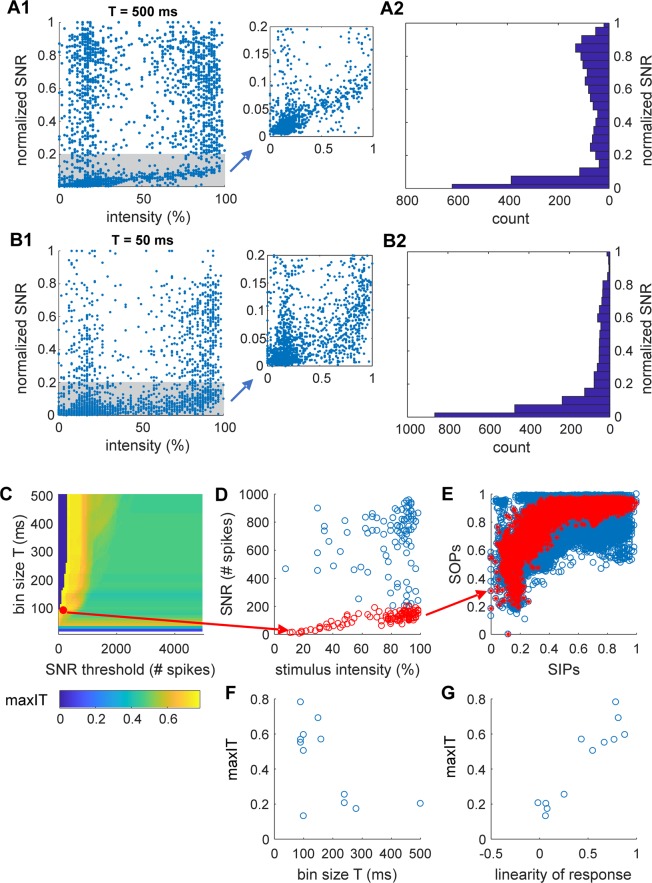
Description of biological network response. (**A,B**) Stimulus intensity and network responses. Statistics of the pooled normalized SNRs (obtained dividing by the max SNR in each experiment) across all the experiments (N = 12) obtained in time bin T of 500 (**A**) and 50 (**B**) milliseconds following the stimulations. (**A1,B1**) Normalized SNRs as function of the stimulus intensity is shown over the all ranges (left) and a zoom of the gray area (where a linearity of response can be observed) is shown on the right plot. (**A2,B2**) Histograms showing the distributions of the responses. Note that although responses are distributed across the all range of response, most of the responses follow within the linear regime highlighted in gray in A1 and B1. (**C–G**) Information processing, response time and linearity of response. The analysis shown in C-G is for a representative experiment (experiment # 9 of Fig. 3). Supp. Fig. 6 report the same analysis for all other experiments. (**C**) Heatmap of the information transmission (maxIT) varying the response bin size T (between 10 and 500 ms in bins of 10) and the threshold on the SNRs (threshold is between 100 and 5000 spikes in bins of 50). (**D**) The maximal information transmission is obtained with T = 90 ms and for a threshold of 200 spikes (red dot in the heatmap of **C**). In the plot of the SNR as function of stimulus intensity, the responses below threshold are shown in red. (**E**) SOPs as functions SIPs (with T = 90 ms) is shown for all responses (blue) and for the ones below threshold (red). (**F**) Maximal information transmission as a function of the bin size T (calculated as shown in C-G) across the 12 experiments. (**G**) Maximal information transmission (same as D) as a function of the linearity of the SNR (which quantify the correlation between SNR and stimulus intensity). Linearity was calculated in the time bin T and SNR threshold optimizing the information transmission.

The information transmission (IT) between the SNN and the BNN was quantified looking at the correlation of the similarity between INPUT pairs (SIPs; see Fig. 4A and Methods) versus the similarity between OUTPUT pairs (SOPs; see Fig. 4C and Methods). The rationale is that in a condition when information transmission take place, two similar INPUT to the BNN should elicit two similar OUTPUT patterns in the BNN. Information transmission was next evaluated across different experiments (N = 12), parameters (response bin size, linearity of network response, average frequency/intensity of stimulation; Figs. 5C–G and 8 top panels) and metrics (entrainment index of the BNN and suppression network synchronization index; Fig. 8 bottom panels). The choice of this simple metric of information transmission, compared to other standard metrics such as the ones derived from information theory (e.g. mutual information), was linked to the impossibility to collect sufficient statistics for each of the stimuli (given the combinatorial number of possible stimuli) in each recording session (lasting between 11 and 40 minutes).

**Figure 6:**
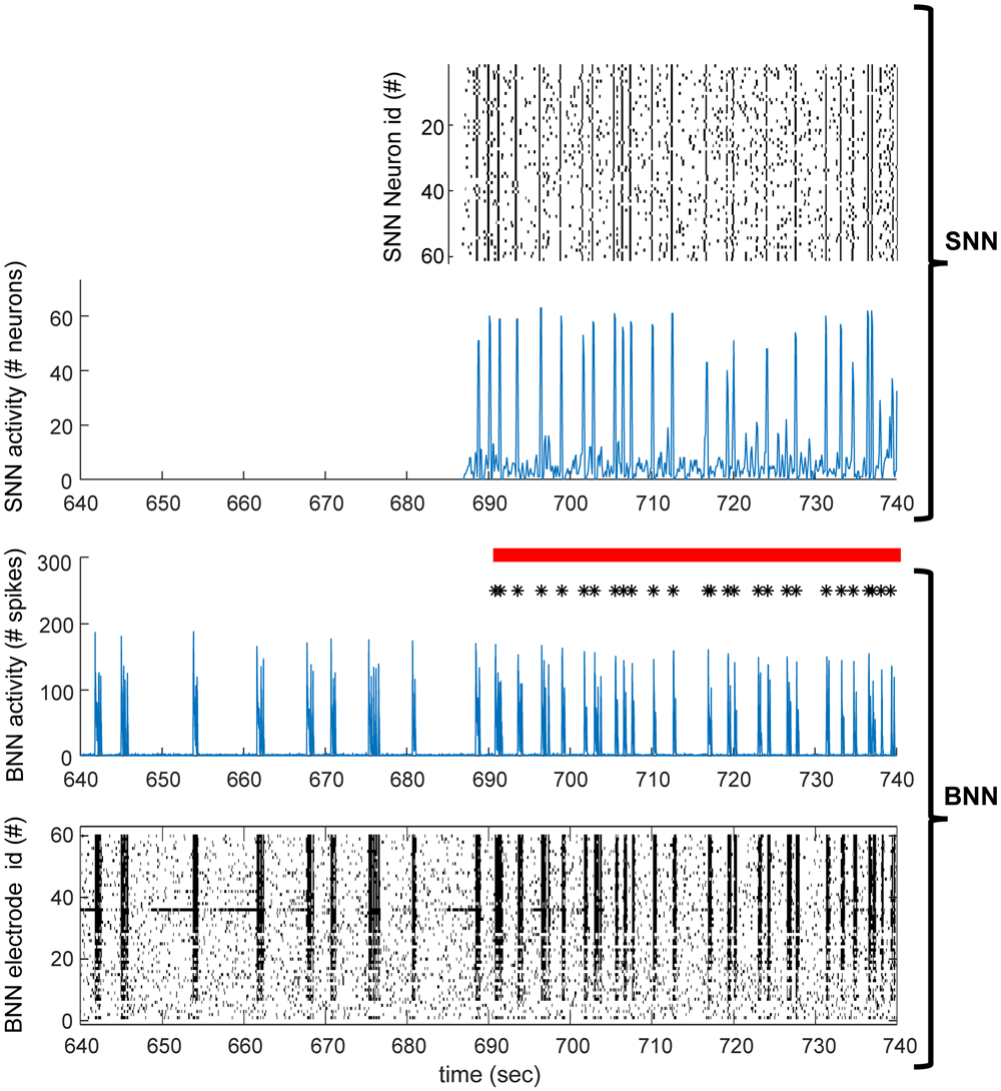
BNN and SNN dynamics. Zoom on a hundred of seconds of activity of the BNN (bottom two plots). After about 50 seconds the communication between the SNN and BNN is switched ON (red horizontal bar) and the timing of the stimuli coming from the SNN are shown (black asterisks), corresponding to synchronized events in the SNN. The raster plot of the activity of the SNN (top) and BNN (bottom) are shown in time bins of a hundred milliseconds. Blue plots represent the total numbers of spikes in the SNN (top) and BNN (bottom) in the in the same time bins of the raster plots. Note the high correspondence between peaks in the SNN and BNN when the communication between the two networks is switched ON.

**Figure 7:**
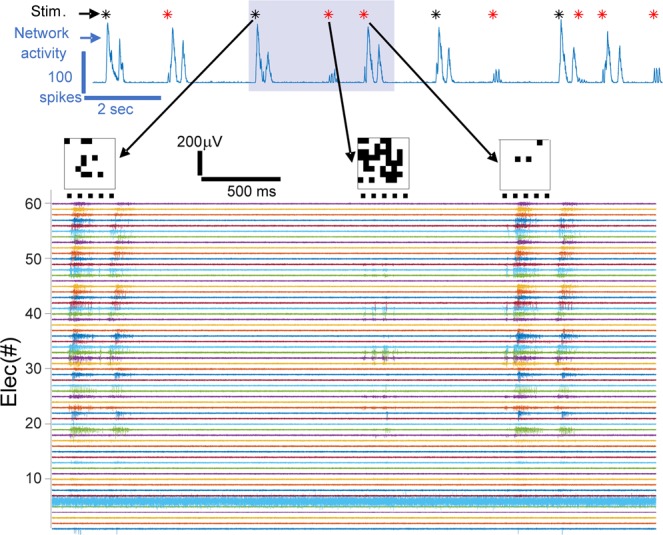
Example of non-linear and linear responses of the BNN to the SNN stimuli. The BNN activity is shown on the top blue plot (total number of spikes recorded in time bins of 10 ms) where asterisks mark the stimulation times. In the case of linear (non-linear) network response the asterisk is red (black). Electrical recordings from all electrodes corresponding to the time window highlighted in gray are shown in the bottom plot. The three stimuli (matrix images) occurring in that time windows are shown on the top of the traces at the time of their occurrence, and the timing of the five pulses of blue light are marked as horizontal black lines under each stimulus-image. Note that in the case of the first stimulation, the response is a network burst and is considered non-linear (it is an overshooting response). The opposite, i.e. linear, case occurs with the second stimulus-image. In the case of the third stimulus-image, although the network respond initially with few spikes and later by generating a network burst, the response of the network in a bin of 90 ms falls within the linear regime.

**Figure 8:**
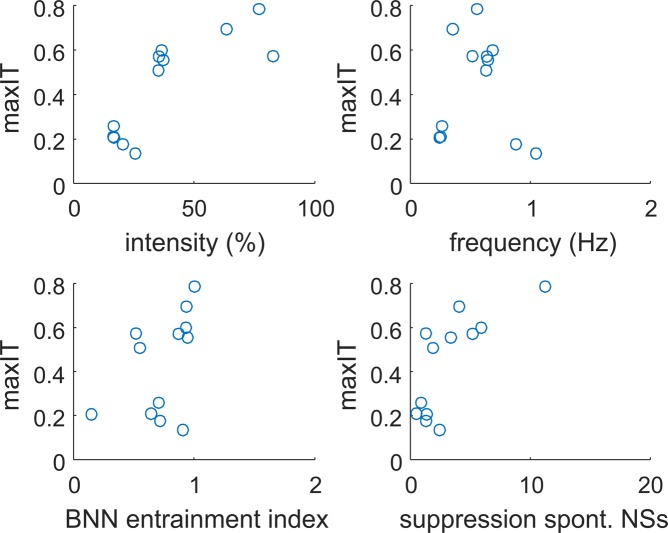
Information transmission across different experimental variables. Maximum information transmission (as calculated in Fig. 6) across all experiments is shown as a function of average stimulus intensity (top left), average stimulus frequency (top right), entrainment index of the BNN (bottom left) and suppression network synchronization index in the BNN (bottom right).

SIPs were measured by the Jaccard coefficient (see Methods; a ratio between the size of the intersection and the union ensembles of the ON matrix elements). Fig. 4A shows the matrix of INPUT similarity for a representative experimental session during which about 200 stimuli were delivered from the SNN to the BNN.

The SOPs were computed based on the evoked vectorial network response of the BNN (Fig. 4B). Specifically, we counted the number of spikes recorded by each electrode within a time window T following the delivery of the SNN stimulus (with T varying between 10 and 500 ms; note that within half a second from a stimulus, both the early and late responses of neurons are typically occurring^31^). The vectorial network response (VNR) for each stimulus was build (Fig. 4B left; each element of the vector corresponds to the number of spikes recorded by corresponding electrode) and the matrix representing the SOPs was calculated (Fig. 4C) using a cosine distance between the VNRs (see Methods). In addition, we also considered the scalar network response (SNR) to each stimulus computed as the sum over the VNR (Fig. 4B right).

We first analysed how the SNR varies as a function of the stimulus intensity (Fig. 5). When the whole time window of response of 500 ms following the stimuli was considered, we observed that the response presented a two-fold behaviour: the SNR was approximately linear to the stimulus intensity with a correlation of 0.70 (N = 12, p < 0.001) when only responses not exceeding 1/8 of the maximal response were considered, while NRs was roughly uniformly distributed above such threshold. Similarly, when a shorter time windows of response was chosen (50 ms) and by focusing on NRs with a threshold below 1/6 of their maximal response, a correlation of 0.57 (N = 12, p < 0.001) between NR and stimulus intensity was observed.

Therefore, for each experiment we determined the optimal time window of response T (in ms, T = N * 10 with N integer varying between 1 and 50, Fig. 5C–E) and the optimal threshold of network response (Fig. 5C and sup. Fig. 6A) maximizing the information transmission between SNN and BNN, i.e. the correlation IT (Information Transmission) between SIPs and SOPs, and we refer to this as the maximal IT (maxIT, sup. Fig. 6B).

Optimal information transmission with a maxIT above 0.5 (0.61 +/− 0.09) was obtained in 8 out of 12 experiments (Fig. 5G and sup. Fig. 6B) with the size of the time bin of response between 80 to 150 ms (116 +/− 31 ms; Fig. 5F) and with a correspondent linearity of the SNR (after optimally discarding above-threshold, i.e. overshooting, NRs) above 0.4 (0.68 +/− 0.15; Fig. 5G).

Next, we looked at how information transmission is related to the stimulus intensity and frequency from the SNN, which are both shaped by the spontaneous network synchronizations displayed by the SNN which mimic those ones occurring in BNNs (Fig. 6).

Information transmission (maxIT) highly correlated (0.81, p-value 0.001) with the average stimulus intensity (Fig. 8) and showed a bell-shaped curve as a function of the average stimulus frequency (Fig. 8), peaked at 0.56 Hz (mean across experiments weighted by maxIT). No significant correlation (p-values > 0.15) was observed with the coefficient of variation (i.e. the standard deviation divided by the mean) of the stimulus intensity and frequency.

Since spontaneous network synchronizations in absence of external stimuli are a very general property of neuronal circuits and networks at multiple spatial scales, both *in-vivo* and *in-vitro*^32,33^, we next looked at the relation between network synchronizations (NSs) in BNNs and information transmission (Figs. 6–8). Spontaneous NSs in the BNNs (in absence of stimuli, i.e. when the communication between SNN and BNN was OFF) occurred with an average frequency of 0.37 +/− 0.08 Hz (SD of the mean, N = 12; average inter-SNSs interval 4.2 +/− 2.7 sec) similarly to what previously reported for cortical cultures^28,29^.

We observed a high correlation (0.73, p-value 0.007) between spontaneous NSs suppression and information transmission (Fig. 8). Spontaneous NSs suppression was quantified as the ratio of the frequency of the BNN spontaneous NSs in basal conditions (i.e. when SNN and BNN were disconnected) versus the frequency of the spontaneous NSs when the communication between the SNN and BNN was switched ON (as an example see Fig. 6 where the activity of BNN and SNN is shown before and after the communication is switched ON). Spontaneous NSs in the presence of stimuli coming from the SNN, were considered as those occurring at least after 500 ms the last stimulus was delivered (Fig. 7).

When looking at the entraining of the BNN, i.e. at the ratio of evoked (within 500 ms from the stimulus) NSs over all NSs when SNN to BNN communication was ON, we observed a high entrainment index of 0.75+/−0.25 when information transmission took place (i.e. with a maxIT above 0.5), although no significant correlation (p-value 0.16) was observed between entrainment index and information transmission.

## Discussion

In this work, we presented the results of the real-time communication between a 100 neuron SNN and a BNN obtained through spatially coded stimuli shaped by the SNN synchronization patterns. The optogenetic stimulation was achieved through spatial (amplitude) light modulation via a DMD mounted on a digital light projector (DLP, i.e. a regular commercially available video-projector). The SNN ran with a millisecond resolution on an FPGA board which embedded a network synchronization detector and a VGA controller to convert synchronized patterns of activity into 800 × 600 pixels images displaying 8 × 8 binary matrices coding for the activity (ON/OFF) of the first 64 artificial excitatory neurons. Once a VGA-image was delivered to the DLP, an additional simultaneous TTL signal from the FPGA board activated the signal generator which controlled the power modulation of the blue light source of the DLP (a high-power blue LED which replaced the original light bulb of the DLP).

The image generated by the DLP was de-magnified (of about fourteen times) through an adapted up-right epifluorescent microscope and focused on the BNN located at the focal plane of the microscope. The BNN, at about four weeks in culture and previously transduced with the fast Channelrhodopsin2 variant ChIEF, responded to blue light stimulation with evoked neuronal firing monitored both by red calcium imaging and multi-electrode recordings.

The SNN and BNN interpedently generated spontaneous dynamics of neuronal activity generating networks synchronizations occurring at similar frequencies. Information transmission from the SNN to the BNN was studied and quantified by measuring the correlation between the similarity of stimuli (incoming from the SNN) versus the similarity of BNN responses (quantified as neural population vectors^34^). Information transmission was studied as a function of the stimulus intensity and frequency, linearity of BNN response, BNN entrainment and BNN spontaneous synchronizations’.

The overall results revealed that information transmission could be achieved if network responses linearly responding to stimulus intensity were considered (linear regime), and therefore over-shooting responses were discarded. In addition, information transmission was optimally achieved when the early response to the stimuli were considered (roughly within the first hundred ms). Over-shooting responses likely represented network synchronizations (or bursts^28^) similar to the one spontaneously generated by the BNN in absence of (or not directly caused by) stimuli. Network bursts stereotypically recruit the entire network, as extensively described in literature in a wide variety of *in-vitro* networks^32^. Information transmission was maximal when the stimuli frequency approximated 0.56 Hz, which is slightly higher but in the same order of magnitude of the intrinsic (spontaneous) frequency of the BNN synchronizations which was about 0.37 Hz. When information transmission took place, we observed high entrainment of the BNN activity and also suppression of spontaneous synchronizations occurring distally (i.e. after half a second) from the last stimulus. Overall these results support the hypothesis that the activity of the BNN needs to be highly entrained by the incoming stimuli from the SNN in order to reliably process it within a linear regime response. We hypothesize that non-linear network responses (over-shooting) could be limited both by optimizing stimulation protocols^35^ and by controlling a sufficiently large portion of the network receiving the input. In fact, in the presented experiments the stimulation was delivered to a very small and spatially defined population of neurons composing the cultured network, where probably a few hundred neurons out of the hundreds of thousands present were stimulated. Therefore, spontaneous synchronizations could also be generated by the “uncontrolled”, i.e. out of the field of view, neuronal population, therefore a complete suppression of burst could not be optimally achieved. In addition, also local optogenetics stimulations could potentially generate a reverberating overall synchronization causing over-shooting non-linear responses in some of the stimuli.

The fact that in our experiments the location of the stimuli (about 800 × 800 micrometers) covers less than one fourth of the region covered by the multi-electrode array (about 1400 × 1400 micrometers) also might have limited the possibility to optimize the read-out of information transmission. The activity recorded in about 4 × 4 electrodes would most likely monitor locally the direct mono- or short-path poly-synaptic responses, while most of the other electrodes would register feedforward reverberating activity. This represent a clear under-sampling issue in the read-out.

We observed a stereotyped relation between SIPs and SOPs in our experiments which tends to cover the upper left part of the plots (Fig. 6 and supp. Fig. 6). This could be also explained by the under-sampling of the neurons used to decode the incoming stimuli. Since always the response of the same neurons is monitored (the neurons being captured by the electrodes), this can necessarily cause a biased similarity between responses also to possibly dissimilar input due to the redundant and small size of the population used for decoding a very large amount of possible stimuli. In addition, spatially proximal, but not strictly similar stimuli could also elicit responses in the same neurons due to their spread arborisations. Therefore, spatial proximity between stimuli could also play a role in causing a higher response similarity.

The trend shown in Fig. 5E and supp. Fig. 6 also highlights the fact that SOP tends to be higher or equal to SIP, possibly reflecting “attractor states”, with convergent responses to input with different overlaps^36^. This effect is particularly pronounced in our experiments when SIP is above 0.2–0.4, and where the attractor’s network state could be represented by a saturated response similar to a global network synchronization.

In this context, these experiments provide important conclusion for the implementation of efficient neuroprosthetic communication, where the high spatial resolution achieved by the stimuli play a key role, and approaching single neuron stimulation could guarantee higher information transmission. In addition, spatial sparseness of stimuli could also be an alternative when stimulations with high spatial resolutions cannot be achieved. Accordingly, overlaps of responses between close neurons could be avoided with a reduction of redundancy of responses and consequent improvement of information transmission.

A similar approach to the one described in this paper has been recently shown^9^ in the context of visual stimuli transduction, where images captured by a camera have been used to stimulate an artificial neural network which in turn was used to shape visual output through a portable DLP which could be used to activate optogenetically downstream neuron in the visual pathway.

In order to optimize information transmission to downstream circuits, our work support the idea of using optogenetically activateable implants which enable spatio-temporal modulation of stimulation patterns where possibly the use of micro-LED over optical fibers might be a more flexible approach^37^.

Advantage of our work is the use of SNN instead of regular stimulation pattern. SNN mimics biological neural activity and then perform biomimetic stimulation. Optogenetic system allows spatial stimulation, SNN adds biomimetic temporal stimulation. Using FPGA platform makes the SNN working in real-time, embedded and low-power. This work provides a real-time adaptive stimulation with a temporal and spatial resolution. Perspective is to create biomimetic optogenetic-based therapeutic solutions for health care.

Future developments will be able to close the loop to enable adaption of SNN activity to biological rhythms. Such a system could be used to investigate neurological disorders using spatial and temporal adaptive stimulation on biological neurons. Furthermore, use of optogenetics as actuator in neuro-prosthetic devices proves advantageous over classical electrical stimulation, as it has been recently demonstrated also in cochlear implants^10^ due to the higher flexibility and selectivity it enables.

## Methods

### Set-up

#### Spiking neural network (SNN) in FPGA

FPGA is a low-power and embedded digital electronic system. It works in real-time and is then well suited for bio-hybrid experiments.

The SNN of this research is composed of Izhikevich neuron model^30^, excitatory and inhibitory AMPA and GABA synapses^38^, short-term plasticity^39^, axonal delay and synaptic noise^40^. All of these SNN components allows a biomimetic dynamic and then provide biomimetic adaptive stimulation to cells. All model parameters are stored in RAM and time step computation is 1 ms. All equations including Izhikevich model are implemented using pipeline technique to ensure high performance. From^41^, simplifications have been performed to optimize number of ressources. Discrete Izhikevich neuron equations are then:

1 $$\begin{array}{c}v[n+1]=1/32\,v{[n]}^{2}+5v[n]+109.375-u[n]+{I}_{b}[n]+{I}_{e}[n]+{I}_{i}[n]\\ u[n+1]=u[n]+a.(b.v[n]-u[n])\end{array}$$

U and v are non-dimensional parameter and v represents the dynamic of membrane voltage. Neuron type is defined by 4 parameters (a, b, c and d). Ib, Ie and Ii represents respectively the bias current, the excitatory and the inhibitory currents from synapses.

AMPA is considered as an excitatory neurotransmitter, which depolarizes the membrane of a neuron, while GABA is considered as an inhibitory neurotransmitter with a hyperpolarization effect. Depolarization or hyperpolarization is represented by a positive or negative contribution to synaptic currents Ie and Ii (Eq. 1). Following the effects of AMPA and GABA, all excitatory or inhibitory synaptic currents tend to exponentially decrease toward zero.

2 $${I}_{syn}[n+1]={I}_{syn}[n]-\frac{1}{t}\cdot {I}_{syn}[n]+{\rm{W}}$$

3 $${\rm{where}}\,{\rm{W}}[{\rm{n}}]={\rm{x}}[{\rm{n}}]\cdot {\rm{W}}$$

4 $${\rm{x}}[{\rm{n}}+1]={\rm{P}}\cdot {\rm{x}}[{\rm{n}}]$$

Synaptic plasticity is defined by 5 parameters (W, x, P and τ). W is the weight of the synapse. X is a scalar factor which indicates the state of the synapse (depression or facilitation). P is a percentage which will be multiplied by the factor x after each emission of a pre-synaptic action potential. If this percentage is larger than 1, this synapse will describe a short-term facilitation. Otherwise, if this percentage is less than 1, this synapse will describe a short-term depression. τ is the time constant of exponential decay (or growth) in facilitation (or depression).

To allow spontaneous activity and make the activity of our network more biologically realistic, we implemented synaptic noise in the current source of the neuron model. We use the Ornstein-Uhlenbeck process which is one of the best model for modeling synaptic noise in a neural network^40^. The Ornstein-Uhlenbeck process is a noise process which is a Gaussian process with bounded variance and which admits a stationary probability distribution.

This SNN is composed of 100 neurons with 7700 synapses. 80% of neurons are excitatory type, 20% inhibitory ones. The number of 100 neurons was chosen to get one to one connection between SNN and an 8 × 8 matrix and is enough number for mimicking dynamic neural activities. The activity of the first 64 neurons (from the 80 excitatory) was converted into an 8 × 8 binary matrix-image (where each element is controlled by a single neuron) used to be projected to the BNN. A VHDL module creates this matrix and handle the VGA communication. Figure 1B. describes the different blocks that compose the FPGA board.

#### VGA module

The 8 × 8 matrix generated by the SNN activity coding for the activity of 64 excitatory neurons, was converted into an image of 800 × 600 pixels (transmitted through VGA to the video projector) and each of the 8 × 8 square had a size of 75 × 75 pixels (768/8 = 96), so covering a part (600 × 600 pixels) of the all images. Out-of-matrix pixels were left dark, i.e. not coding any information. Square images are sent by VGA to the digital light processing (DLP) projector (video-projector). The video-projector generates images through a Digital Micromirror Device (DMD) of 1024 × 768 mirrors, i.e. pixels. A custom VHDL designs converted the spiking neuronal signal into analog signals which is sent to the projector. Sup. Fig. 2 shows the simplified principle of the system that connect neurons to the projector which reproduce the neuronal activities through light. The SNN sends spike timing to a burst detector module that creates an enable signal to the LED depending the number of spikes that occurs in less than 10 ms. SNN spikes are converted in a square 8 × 8 where each square corresponds to one neuron. If one neuron spikes, his square becomes white, if not it stays black.

#### Video-projection of images and optical set-up

The light intensity at the microscope focal plane (the location of the BNN) was controlled spatially via amplitude modulation of a collimated beam and temporally via a TTL trigger to the STG1008 (Multi-channel System) stimulator, as shown in Fig. 1C. A collimated beam of 460 nm light was produced using a powerful LED (Luminus PT-120-B) and coupled to a Texas Instruments Digital Micromirror Device (DMD) driven by a commercial SHARP XR-10Xprojector. The video-projector was modified to allow operation without the original light source and color wheel. Optics other than the final expander lens of the projector were left in place, creating an image directly at the focal length of the coupling lens to the microscope. The power at the focal plane was 5–10 mW/mm^2^. The images from the video-projector were refreshed at a rate of 60 Hz. The TTL pulse from the FPGA board activated the light stimulation protocol run by the STG1008 stimulator, which was composed of five pulses of 3.75 Volt lasting 30 ms each separated by 40 ms of dark (0 Volt). The STG1008 stimulator output was controlling the custom-made LED power driver. We used 75% of the maximum LED power which guaranteed a sufficient power at the focal plane of the microscope. Since the power used was just enough to stimulate neurons on the illuminated regions, we do not expect any neurons to be stimulated by the scattered ambient light of the projector out of those regions.

The LED was driven using a custom-made switch mode power supply with adjustable current and voltage limiting capable of providing up to 40 A at 5 V as well as a 0–5 V input for external control of the output power. The circuit was based around a Texas Instruments TL494 chip.

### Recordings

#### Primary cell culture

As experimental model for this research, primary cortical cultures from embryonic rats have been used. All animal care protocols, and all experimental protocols used for this study were conducted according to the animal research guidelines from Tel Aviv University and were approved by the Tel Aviv University Animal Care Committee. Cultures were prepared as described previously^29,42^. Briefly, the entire neo-cortex of P0-1 mouse was removed, chopped with scissors in a Trypsin EDTA solution. The cortical tissue was digested witha papain-based dissociation buffer (2.5 mM CaCl_2_, 0.83 mM EDTA, 137 U papain (Sigma-Aldrich)), 100 μl DNAse (Sigma-Aldrich), 3–5 crystals of L-Cysteine (Sigma-Aldrich), HBSS with 20 mM HEPES (pH 7.4) and placed on a rotating shaker for 15 min at room temperature for mechanical dissociation by trituration. Cells were resuspended in modified essential medium (MEM) without L-glutamine with essential amino acids (Beit Haemek, 06-1025-01-1A), 5% heat-inactivated fetal calf serum (Biological Industries), heat-inactivated 5% horse serum (Beith Haemek, 04-004-1), 2 mM glutamine (Beit Haemek, 03-020-1c), 3 mg/ml glucose, 2% B-27 (Gibco, 17504-044), and 0.5% Pen/Strep (100 U/ml penicillin, 100 μg/ml streptomycin; Beit Haemek, 03-031-1B), and plated on poly-D-lysine (PDL, Sigma, catalog no p-7889) covered multielectrode arrays (500/30iR-Ti or HD 30/10iR-ITO, by Multi Channel Systems) with a cell density of 3000–4000 cellsmm−2 (∼1.5 × 106 cells per dish). Cultures were maintained at 37 °C with 5% CO_2_. Growth medium was partially replaced every 3–4 days (MEM-EAGLE (without L-glutamine with essential amino acid), 5 mg/ml glucose, 5% heat-inactivated fetal calf serum, 0.8% GlutaMAX (100 × ; Gibco, 35050–038), 0.5% Pen/Strep, 2 mM glutamine, 2% B-27). At 7 DIV, cultures were transduced with AAV2/1-hSyn-oChIEF-mCitrine vectors (assembled in HEK293T cells using a previously published protocol^43^).

#### MEA recordings and calcium imaging

Before recording the activity of the neuronal cultures grown on the MEAs, the cultures were loaded with the RFP calcium sensor RHOD-3 (Life Technologies). The loading procedure and recording set-up was similar to what described by Bonifazi *et al*., 2013 and Kanner 2018. Briefly, in order to load the cells with the calcium-sensitive dye, cultures were incubated for 40 min in 1 ml buffered-ACSF solution (containing, in mM, 10 HEPES, 4 KCl, 1.5 CaCl_2_, 0.75 MgCl_2_, 139 NaCl,10D-glucose, adjusted with sucrose to an osmolarity of 325 mOsm, and with NaOH to a pH of 7.4) supplemented with 1 μl of 10% pluronic acid F-127 (Biotium 59000) and 1 μl RHOD-3 previously diluted in 1 μl anhydrous DMSO. Following incubation, cultures were washed, incubated for another 30 minutes with buffered-ACSF and next transferred to set-up and recorded in a fresh buffered-ACSF at 37 °C in an open-air environment. In order to avoid artifacts due to evaporation and pH changes, the buffered-ACSF was replaced after each recording session lasting between 20 and 40 minutes.

Calcium-fluorescence images were acquired with an EMCCD camera (Andor Ixon-885) mounted on an Olympus upright microscope (BX51WI) using a 10X water immersion objective (Olympus, LUMPLFL NA 0.4). Images were acquired at 57 frames per second in 2 × 2 binning mode (composed of 501 × 502 pixels out of the 10001 × 1002 pixels of the full EMCCD chip) using Andor software data-acquisition card (SOLIS) installed on a personal computer, spooled to a high capacity hard drive, and stored as uncompressed multi-page tiff file libraries.

Fluorescent excitation was provided via a 120-W mercury lamp (EXFO x-cite 120PC) coupled to the microscope optical axis with a dichroic mirror and equipped with an excitation filter matching the dye spectrum (Chroma dsRed 41035). A U-DP beamsplitter (Olympus) equipped with a long pass dichroic mirror (cut-off 532 nm) was placed between the eyepiece and the filter-cube wheel of the microscope and it was used for calcium imaging with the red sensor (RHOD-3). Red calcium imaging also required the relocation of the emission filter of the dsRed-cube above the long-pass dichroic mirror. In this way, the blue light used for ChIEF excitation entering through the U-DP coukld reach with full power the neuronal sample while it was filtered and attenuated by the dsRed emission filter before reaching the camera providing optimal conditions to record red calcium fluorescence. However, given the high power of the blue LED used for optogenetics, residual artefact blue light shined during the stimulations, could reach the camera and therefore it was used to verify and record the spatial-temporal features of the stimuli. Regular GFP imaging was performed normally in absence of the long pass dichroic mirror.

The commercial system purchased from Multi Channel Systems (MCS, Reutlingen, Germany) has been used for multi-electrode array recordings. We used standard MEAs consisting of 59 round electrodes made of TiN, where electrodes are equidistantly positioned in an 8 × 8 layout grid, with an inter-electrode distance of 200 µm and a microelectrode diameter of 30 µm. A ground electrode embedded on the MEA was used. For this study, we recorded and studied the activity of networks between 21 DIV to 28 DIV.

#### Whole-cell recordings

In order to test the response of the neurons to blue light stimulations, celle neurons were grown on ceovrslips and not on MEAs. Recordings from cultured neurons were performed under similar conditions as previously described. Cells which expressed the ChIEF construct were identified by mCitrine fluorescence and were patched using a borosilicate glass electrodes (4–6 MΩ resistance) filled with (in mM): 110 potassium gluconate, 10 EGTA, 20 HEPES, 2 MgCl_2_ and 10 glucose; pH 7.3 (adjusted with KOH) and 280–290 mOsm. In order to isolate currents associated with ChIEF activity, inhibitory and excitatory transmission were blocked using 1 mM picrotoxin and 25 mM NBQX (Tocris). Cells were optically stimulated using a 200 µm thick optic fiber (Prizmatix) connected to a blue LED and their responses to three stimulation frequencies was recorded (20 bursts of 5 stimulations, 5 ms each, at 25, 12.5 and 7 Hz, with an inter-burst interval of 20 s). Failure rates were calculated as the percentage of failed action potentials out of the total amount of stimulations delivered at each frequency. Results are shown in suppl. Fig. 7.

#### Immunocytochemistry

A different set of cortical cultures plated on MEAs and transduced at 7 DIV with AAV2/1-hSyn-oChIEF-mCitrine vectors, were used for immunostaining at 21 DIV. Cells were washed with PBS and fixed with 4% PFA for 10 min at room temperature. Next, cells were permeabilized with 0.5% Triton x100 (Sigma-Aldrich) in PBS for 10 min and blocked in blocking solution (2% BSA, 10% normal donkey serum, and 0.25% triton x100 in PBS) for 1 h at room temperature. The cultures were incubated overnight with the primary antibodies (mouse- anti-NeuN, 1:200, Millipore Cat# MAB377) at 4 °C, then washed three times with PBS and incubated with matching secondary antibody (1:400, Alexa Invitrogen) for 1 h at room temperature. Cultures were washed twice with PBS and incubated with DAPI (1:1000, Jackson) in PBS for 10 min. Cultures were further washed twice with PBS.

### Data analysis

All data analysis has been performed using MATLAB (MathWorks, Natick, MA). In the whole paper, reported errors correspond to standard deviations.

#### INPUT to the BNN

The similarity between input pairs (SIPs), i.e. between the binary 8 × 8 INPUT images, was computed using the Jaccard similarity index, which is calculated as the ratio between the number elements which are shared between both images (cardinality of intersection ensemble) over the total number of elements in both images (shared and un-shared, i.e. cardinality of union ensemble).

The intensity of the 8 × 8 image input was quantified as the number of “ones” elements set to one ivided by 64 and multiplied to 100 in order to normalize it between 0 and 100%. The frequency of input was calculated as the ratio of the mean inter-stimulus interval, the latter expressed in seconds. Note that in the all work, since as each single stimulus is composed of five light pulses (30 ms ON and 40 ms OFF), the time t of a stimulus was considered as the time of the starting of the first pulse.

#### BNN activity signals and metrics

Electrical recordings were first pre-processed using a zero-phase digital filtering (“filtfilt” function) with high-pass cut-off set at 100 Hz. All analysis steps described below were applied to the filtered signals.

For each electrode, the noise level was estimated by fitting the probability density function of the signal with a Gaussian, in the 5th to 95th percentile interval, over an entire recording. A threshold of 4 standard deviations below the mean was used to extract the timing of the negative peak of the spikes with an imposed refractory time between spikes of 1 ms. In this work, no spike sorting was applied and all the spikes, i.e. multi-unit activity, recorded by each electrode were considered.

Extraction of the calcium imaging signal for each single cell was performed using the previously described procedure for cell body identification^2,29,44^.

The Vectorial Network Response to a given stimulus delivered at time *t* (VNR(t)) for a time bin of response T was calculated as a the number of spikes each electrode was recording in the time interval [t,t+T]. So, the VNR was a vector of 59 elements, where each element corresponded to an electrode.

The Scalar Network Response (SNR) was calculated as the sum of the VNR over all electrodes.

The time window T of response varied between 10 and 500 ms in bin of 10 ms.

The similarity between output pairs (SOPs), was quantified as one minus the cosine distance between the VNRs.

The correlation between between the SIPs and the SOPs (correlation of similarity) was used as metric of information transmission (IT) between the SNN and the BNN.

In the paper, when we refer to correlation coefficient, we refer to the Pearson correlation coefficient.

In order to identify the optimal conditions to maximize information transmission and calculate the maximal IT (maxIT), given the set of stimuli within a given experiment, we calculated the correlation between SIPs and SOPs both by varying T (between 10 and 500 ms in bin of 10) and the subset of stimuli included in the calculation by discarding overshooting SNRs according to what follows. After normalizing the SNRs between zero and one by dividing the SNRs to its maximal value (nSNRs), a threshold to the nSNRs between 0.02 and 1 (varying in bins of 0.02) was applied and only stimuli below that threshold were used to calculate the SIPs vs SOPs correlation. In order to guarantee a minimal statistic, cases when more than the 75% of the whole stimuli exceeded the threshold were discarded. The linearity of response was calculated as the correlation between SNRs and intensity of stimulation on given a subset of stimuli after thresholding.

Network synchronizations (NSs) were identified from the instantaneous scalar network activity (iSNA). The iSNA was calculated by counting all spikes recorded in the network in sliding time windows of 10 ms (with sliding step of 1 ms) over an entire recording session. Peaks of iSNA with a minimal threshold of 25% of the maximal iSNA and identified with a refractory time of 100 ms, were considered as NSs. The time of the maximum of the peak of iSNA was used as time of the NS.

The BNN entrainment index was calculated as the ratio of evoked versus spontaneous NS during the epoch when the communication between SNN and BNN was switched ON. Evoked (spontaneous) NS were marked as the ones occurring within (after) the 500 from the last stimulus. The suppression of spontaneous NSs index was calculated as the ratio between the frequency of spontaneous NSs in the BNN in the epochs preceding and when the communication with the SNN was switched ON. The frequency of the NSs was calculated as one over the average inter-NS interval (the latter expressed in seconds).

## Supplementary information

Supplementary information with supplementary figures.
