# Supplementary material for: Toward neuroprosthetic real-time communication from in silico to biological neuronal network via patterned optogenetic stimulation
Source: Sci Rep. 2020 May 5;10:7512. doi: 10.1038/s41598-020-63934-4 (PMC7200763; doi:10.1038/s41598-020-63934-4)
Supplement: Supplementary file 1 — Supplementary information with supplementary figures. [file 41598_2020_63934_MOESM1_ESM.pdf]

Toward neuroprosthetic real-time communication from in silico to biological neuronal network via patterned optogenetic stimulation

Yossi Mosbacher[1]**, Farad Khoyratee[2]**, Miri Goldin[10]**, Sivan Kanner[3]**, Yenehaetra Malakai[4], Moises Silva[5], Filippo Grassia[6], Yoav Ben Simon[7], Jesus Cortes[9,10], Ari Barzilai[3,8]*, Timothée Levi[2,11,12]*, Paolo Bonifazi[9,10]*

Affiliations

[1]Department of Particle Physics and Astrophysics, Weizmann Institute of Science, Rehovot, Israel;
[2]IMS, University of Bordeaux, France
[3]Department of Neurobiology, George S. Wise, Faculty of Life Sciences, Tel Aviv University, Tel Aviv 69978, Israel
[4]IUT GEII, University of Bordeaux, Talence, France
[5]Faculty of Informatics, University of the Basque Country, San Sebastian, Spain
[6]University of Picardie Jules Verne, Laboratory of Innovative Technologies, Amiens, France
[7]Institute of Science and Technology (IST) Austria, Klosterneuburg 3400, Austria
[8]Sagol School of Neuroscience, Tel Aviv University, Tel Aviv 69978, Israel.
[9]IKERBASQUE: The Basque Foundation for Science. Bilbao, Spain
[10]Biocruces Health Research Institute. Barakaldo, Spain
[11]LIMMS/CNRS-IIS, the University of Tokyo, Tokyo, Japan
[12]IIS, the University of Tokyo, Japan
****Contact information of equally contributed senior authors:** Dr. Paolo Bonifazi; email : paol.bonifazi@gmail.com; Dr. Timothée Levi, email : timothee.levi@u-bordeaux.fr*
*****Equal first author contribution***

Abstract

Restoration of the communication between brain circuitry is a crucial step in the recovery of brain damage induced by traumatic injuries or neurological insults. In this work we present a study of real-time unidirectional communication between a spiking neuronal network (SNN) implemented on digital platform and an in-vitro biological neuronal network (BNN), generating similar spontaneous patterns of activity both spatial and temporal. The communication between the networks was established using patterned optogenetic stimulation via a modified digital light projector (DLP) receiving real-time input dictated by the spiking neurons' state. Each stimulation consisted of a binary image composed of 8x8 squares, representing the state of 64 excitatory neurons. The spontaneous and evoked activity of the biological neuronal network was recorded using a multi-electrode array in conjunction with calcium imaging. The image was projected in a sub-portion of the cultured network covered by a subset of the all electrodes. The unidirectional information transmission (SNN to BNN) is estimated using the similarity matrix of the input stimuli and output firing. Information transmission was studied in relation to the distribution of stimulus frequency and stimulus intensity, both regulated by the spontaneous dynamics of the SNN, and to the entrainment of the biological networks. We demonstrate that high information transfer from SNN to BNN is possible and identify a set of conditions under which such transfer can occur, namely when the spiking network synchronizations drive the biological synchronizations (entrainment) and in a linear regime response to the stimuli. This research provides further evidence of possible application of miniaturized SNN in future neuro-prosthetic devices for local replacement of injured micro-circuitries capable to communicate within larger brain networks.

Supplementary Figures

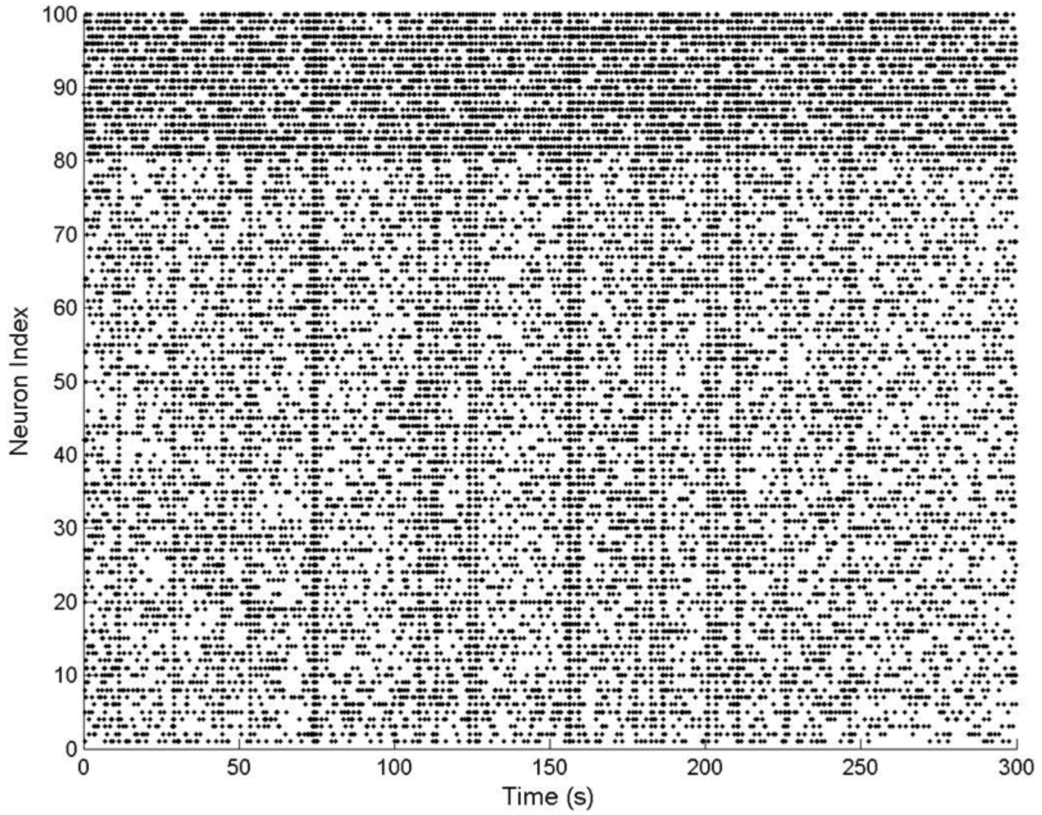

Sup. Fig. 1. Raster plot of the SNN2. *The time resolution of computation is 1 ms. The first 80 neurons are excitatory, the 20 other neurons are inhibitory. This SNN includes 100 Izhikevich neurons, AMPA and GABA synapses, axonal delay, synaptic noise and short-term plasticity. This SNN on FPGA works in real-time.*

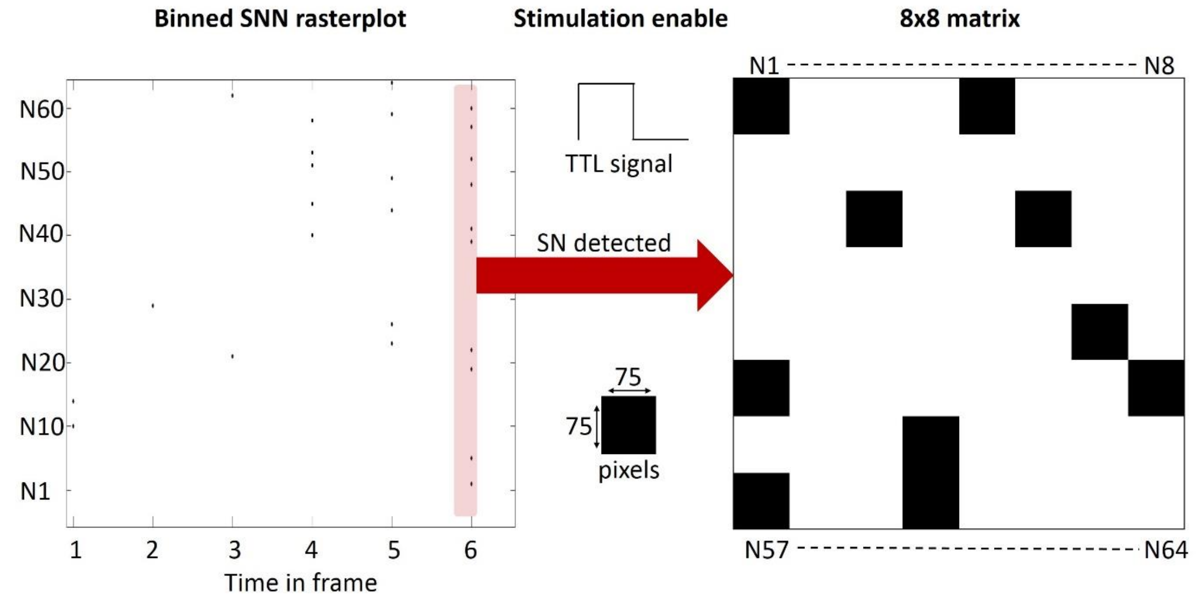

Sup. Fig. 2. Description of the conversion from binned SNN raster plot to 8x8 matrix image. *From SNN rasterplot, a network synchronization detector module compute in a time windows (100ms or 200ms) the number of synchronous spikes. If this number is over one threshold (10, 7, or 4 depending*

experiments), a TTL signal is sent to the STG stimulator and a 8x8 matrix image is generated which is the image of the neuron spikes. In this example, 10 neurons are spiking during this network synchronization (N1, N5, N19, N22, N39, N41, N48, N52, N57 and N60).

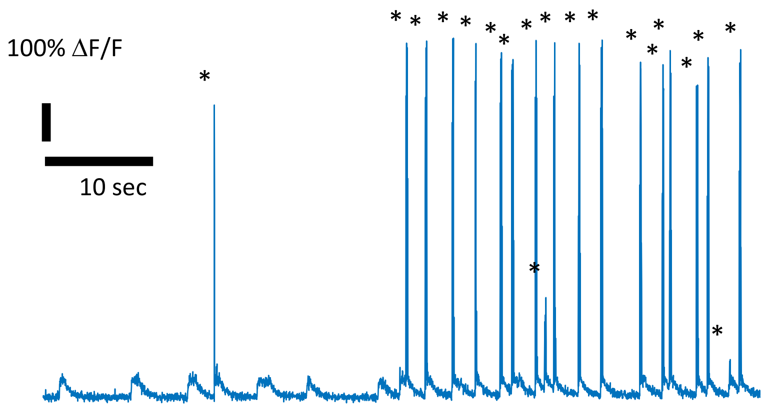

Sup. Fig. 3. Calcium imaging. *Calcium trace of a representative neuron. Asterisks mark light artefact due to the video-projected stimuli. The size of the artefact depends on the distance between the stimulated spot and the location of the neuron. Note that the neuronal activity is synchronized to the light-stimuli. In absence of light stimuli, spontaneous neuronal dynamics are recorded.*

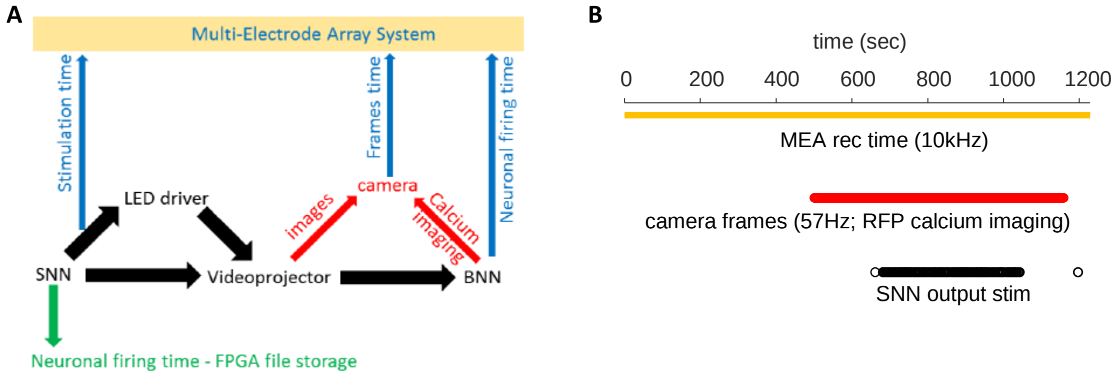

Sup. Fig. 4. Devices' synchronization and cross-device time envelope for a representative experiment. A. *Scheme of the synchronization between devices. The analog input of the MEA system allowed to record the stimulation times (SNN), frame time (camera) and neuronal spikes (MEA).* ***B.*** *For a representative experiment, yellow mark the epoch when the MEA system is recording, red when the camera is recording and black when the communication between SNN and BNN is switched ON.*

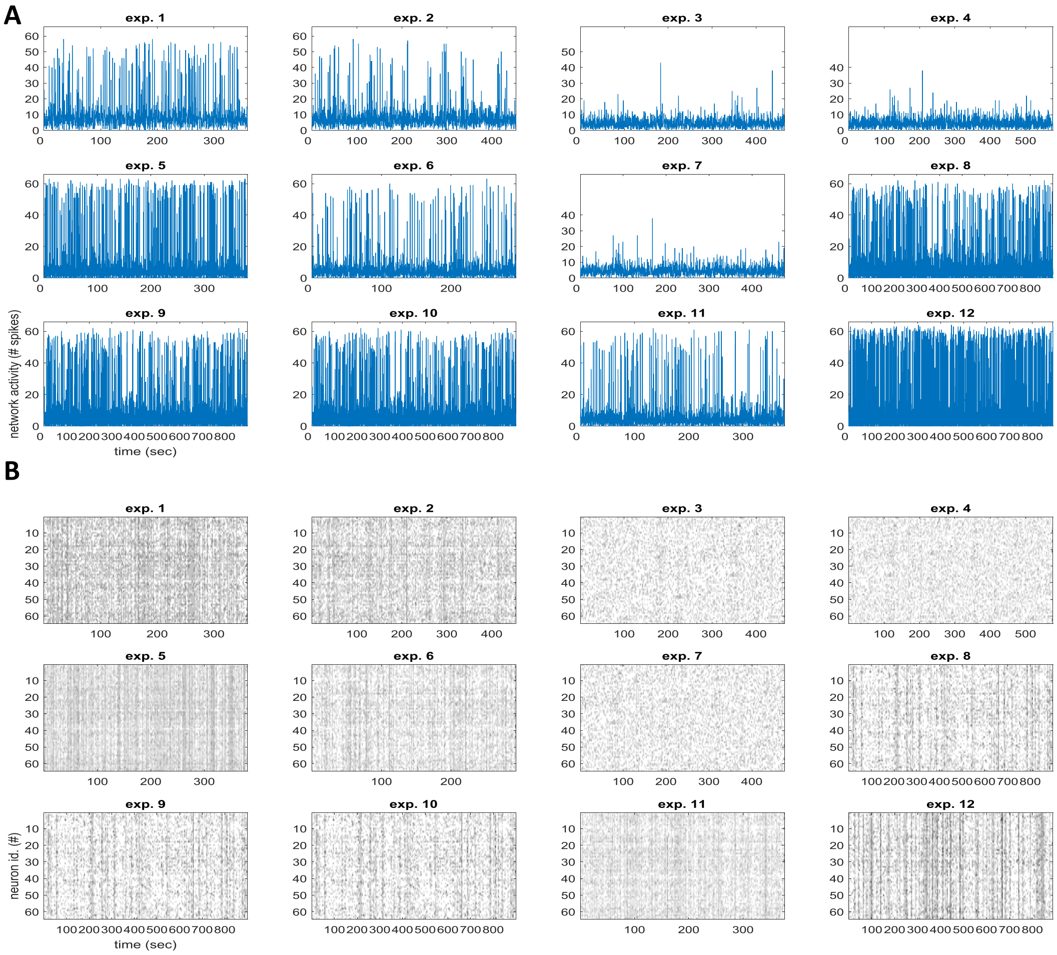

Sup. Fig. 5. Network synchronizations and raster plots of the different SNN. A *total number of spikes recorded in each time bin (see time of computation in table 3) for each of the 12 SNN used in this work. B raster plot of the SNN in the same time bins.*

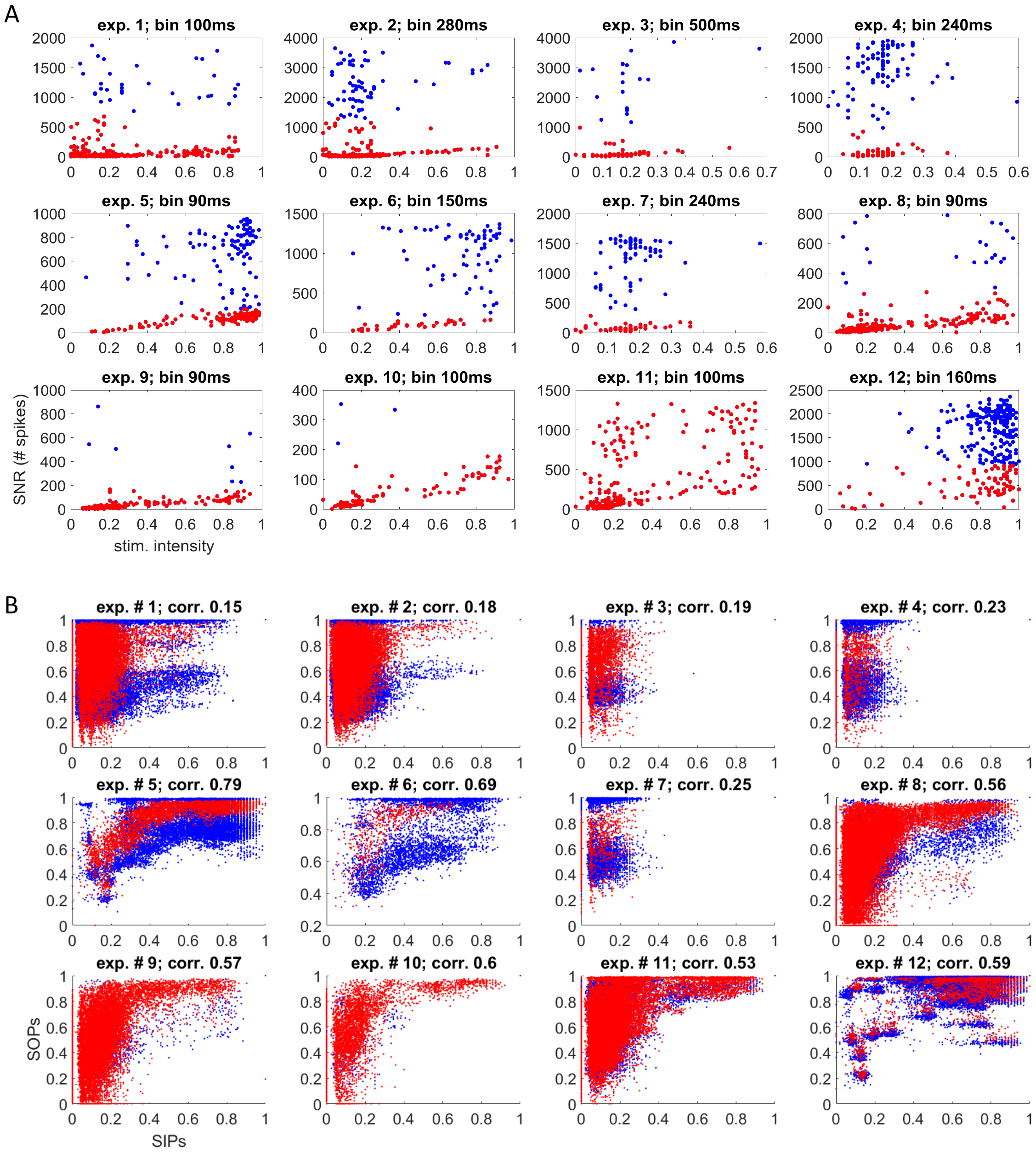

Sup. Fig. 6. BNN and SNN dynamics for all experiments. ***A*** *scalar network response as a function of the stimulus intensity for each experiment. The time bin reported on the top of each plot is the one optimizing the information transmission in conjunction with the use of stimuli not exceeding an optimal threshold where the response follows a linear trend (red dots mark the stimuli below threshold).* ***B.*** *Information transmission is quantified as the correlation between SIPs and SOPs. For each experiment is reported the maximal correlation obtained across bin size and threshold (see panel A). Red dots show the SIPs/SOPs for the selected stimuli/responses identified (in red) in panel A.*

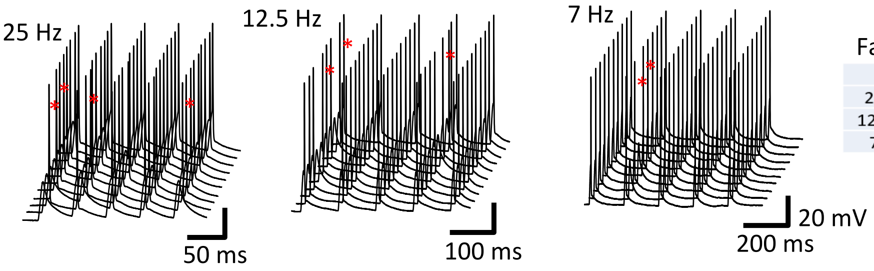

Failure rates (%) (N=9 cells)

	Average	SEM
25 Hz	3	0.931695
12.5 Hz	2.625	0.686725
7 Hz	1.5	0.426401

Sup. Fig. 7. ChIEF failure rates in the experimental design. A. Summary of action potential failure rates during bursts consisting of 5 optogenetic stimulations, 5 ms each, delivered at 25, 12.5 and 7 Hz (N=9 cells). **B.** Representative traces in current clamp from one cell to the three activation frequencies. Action potential failures are denoted using red asterisks.
